# Contribution of Aging, Obesity, and Microbiota on Tumor Immunotherapy Efficacy and Toxicity

**DOI:** 10.3390/ijms20143586

**Published:** 2019-07-23

**Authors:** Regina E. M. Baiden-Amissah, Sandra Tuyaerts

**Affiliations:** 1 Division of Gynecologic Oncology, Department of Oncology, KU Leuven, 3000 Leuven, Belgium; 2 Leuven Cancer Institute (LKI), 3000 Leuven, Belgium

**Keywords:** cancer immunotherapy, aging, obesity, gut microbiome, biomarker

## Abstract

Cancer immunotherapy has entered the forefront of cancer treatment, but major challenges still exist, such as the limited proportion of patients that respond to treatment and treatment-related toxicity. Therefore, biomarkers to predict which patients will benefit from therapy without major side effects are of the utmost importance. Moreover, novel therapeutic targets to increase the proportion of responding patients on a given immunotherapy or to alleviate immunotherapy-induced toxicity could be a valuable adjunct to immunotherapy treatment. Host factors such as age, obesity, and the composition of the gut microbiome have considerable effects on immune responses and, hence, could have a large impact on the outcome of immunotherapies. Moreover, since these host factors differ considerably between preclinical mouse models and human cancer patients, it might be possible that these host factors account, in part, for the observed discrepancies in outcomes between mice experiments and clinical trials. In this review, we discuss the latest data on the influence of aging, obesity, and the gut microbiome on the anti-tumor immune response and immunotherapy and propose avenues to increase our knowledge on this topic in order to improve patient selection for cancer immunotherapy treatment.

## 1. Introduction

Tumors are considered to be antigenic due to the presence of altered proteins resulting from genomic alterations, thereby making them non-self to the immune system. Hence, most transformed cells or developing cancers are likely to be rejected spontaneously by the immune system. However, certain factors such as tumor-driven inflammation, immunoediting, and immune dysfunction affect the capacity of the immune system to target cancer cells, ultimately leading to the outgrowth of non-immunogenic clinically relevant tumors [1]. The increased knowledge of the interactions between cancer and the immune system has led to the development of immunotherapeutic approaches in the past few decades. The success of immune checkpoint blockade (ICB) in various tumor types has been a major breakthrough for the treatment of cancer and has boosted the cancer immunotherapy research field [2,3].

However, the increasing experience with this type of treatment also showed that immunotherapy is only effective in a subset of patients and can be accompanied by side effects that can be quite severe. This awareness created a boost in research aiming to identify biomarkers for better patient selection for cancer immunotherapeutic approaches. Since cancer cells interact continuously with their surrounding host tissues, it is clear that host factors, for example, age, obesity, and the composition of the microbiome, can influence the immune response and can impact the effectiveness of immunotherapy.

In this review, we focused on unraveling the delicate balance between age, obesity, cancer, immunity and the gut microbiome to reveal how these host factors impact the efficacy and toxicity of cancer immunotherapy.

## 2. Effect of Host Factors on the Immune System

### 2.1. Age

Cancer is an age-related disease with more than 50% of patients being older than 65 years. Due to the increase in life expectancy, the elderly population has increased and it is projected that the number of cancer patients and mortality in the elderly will increase as well [4,5,6].

**Figure 1:**
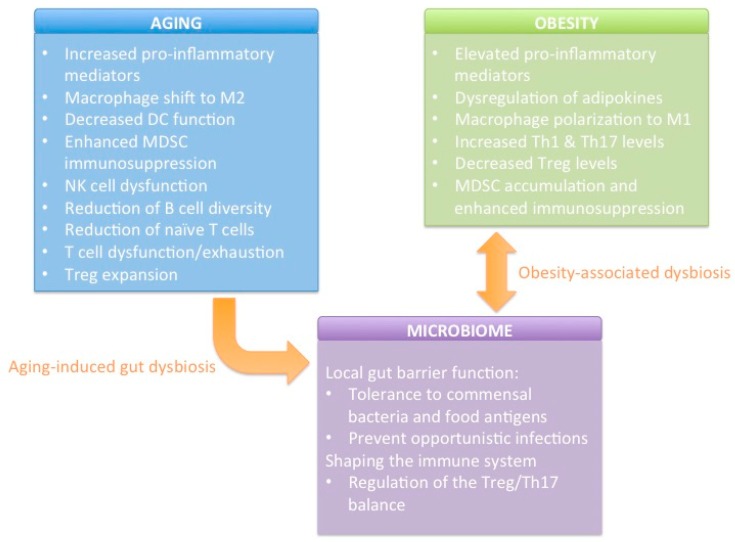
The immunological effects of aging, obesity, and the microbiota. DC: dendritic cell; MDSC: myeloid-derived suppressor cell; NK: natural killer cell; Treg: regulatory T cell; Th: T helper cell.

Age-related changes in immune response, characterized by progressive immunodeficiency, are collectively called “immunosenescence” [7,8,9,10]. Understanding the mechanisms of immunosenescence will help to limit age-related decreases in immunity or prevent its consequences if possible. In general, upon aging, immunity is tilted towards the innate immune response and induces a state of chronic low-grade inflammation (Figure 1). This is characterized by increased levels of systemic inflammatory molecules, such as C-reactive protein, IL-6, TNFα, IL-1β, CCL7 (MCP-3), and Lymphotoxin alpha, and altered functionality of macrophages (shift towards the M2 phenotype) [11,12], dendritic cells (DCs) (decreased phagocytic uptake and migration) [13,14], myeloid-derived suppressor cells (MDSCs) (increased immunosuppression) [15,16,17,18], and NK cells (altered distribution and reduced cytotoxicity) [19]. Concerning the adaptive immune response, aging causes a reduction in B cell levels [19], a decreased diversity of the B cell repertoire [20,21,22], reduction in the naïve T cell repertoire, T cell dysfunction, T cell exhaustion, and regulatory T cell (Treg) expansion [8,23,24,25,26,27,28].

The immunosenescence-associated decline in naïve T cells might be important for neoantigens (from genomic alterations or viral infection) that are “new” for the immune system and to which no memory T cells are present. This might create a “hole in the anti-tumor T cell repertoire” in elderly patients. It is currently unknown if such a “hole in the anti-tumor T cell repertoire” exists, but this phenomenon could be remediated by the use of engineered T cell receptor (TCR) T cells. In this concept, the appropriate TCR genes would be cloned, inserted into the patient’s T cells, and re-infused to ‘‘fill the hole’’ [7].

Altogether, these processes have a large impact on immunity that could hamper the effectiveness of cancer immunotherapy.

### 2.2. Obesity

Incidences of obesity have been rising drastically over the last decades and is a major risk factor for various types of cancer, especially colon cancer, renal cancer, breast cancer, and endometrial cancer. Furthermore, obesity also increases tumor progression [29]. 

Obesity is associated with a state of chronic inflammation, leading to immune senescence similar to aging, hence, also called inflammaging (Figure 1). The inflammatory state is driven by the adipose tissue through secretion of soluble factors that recruit immune cells into the adipose tissue, consisting mainly of macrophages and T cells. Several soluble factors contribute to the obesity-associated inflammation, such as proinflammatory cytokines (IL-6, PGE_2_ and TNFα), chemokines (CCL2, CCL5), and dysregulation of adipokines (increased leptin and decreased adiponectin). The overall effect is macrophage polarization to the M1 phenotype, increased Th1/Th17 cells and decreased Treg levels [30,31]. Furthermore, Treg present in adipose tissue display a highly restricted TCR repertoire, suggesting they recognize certain autoantigens in the adipose tissue. However, the exact autoantigen(s) recognized by adipose tissue-associated Treg remain to be identified [32].

Recently, it has also been shown that the chronic inflammatory milieu of adipose tissue induces the accumulation of MDSCs, both the monocytic (M-MDSC) and the polymorphonuclear (PMN-MDSC) subsets. Accumulation of MDSC is driven by leptin through the induction of IL-1β, TNFα, and IL-6. The cytokines IL-1β and TNFα also induce leptin, thereby creating a feedback loop that sustains the inflammatory environment [33]. Hence, inflammasomes that produce IL-1β are hypothesized to be key regulators of the proinflammatory adipose tissue environment. Myeloid-derived suppressor cells themselves further increase the accumulation of adipocytes [34]. Obesity-induced MDSCs show an enhanced potency and promote tumor growth by suppressing tumor-specific T cells and preventing their entry into the tumor microenvironment (TME). Leptin also plays a role in the increased potency of MDSCs, since leptin induces IFNγ that regulates PD-L1 expression by MDSCs and is one of the mechanisms by which MDSCs suppress T cells [35]. 

### 2.3. Microbiome

The human microbiome is defined as the collective genomes of microbes that live symbiotically on and within various sites of the human body of which the gut harbors the largest number of microbes. The gut barrier functions as the interface between these microbes and the host. Of all microbes residing in the gut, four major phyla (Firmicutes, Bacteroides, Proteobacteria, and Actinobacteria) account for 98% of the microbiota. External factors and host factors can induce disruption of the delicate composition of the microbiome of the gut (gut dysbiosis). The most well-known factor able to disturb the gut microbiota composition is the use of broad-spectrum antibiotics [36]. 

It is obvious that the crosstalk between microbiota and the immune system is critical and allows for tolerance to commensal bacteria and food antigens and enables the immune system to prevent opportunistic infections [37]. However, in recent years it became clear that in addition to this local immune response, the microbiota exerts broader effects on innate and adaptive immunity and help to shape the immune system as a whole (Figure 1) [38]. This concept was noted via the use of germ-free mice that lack intestinal microbiota and have severe immune defects. Disruption of the gut barrier function increases gut permeability to microbes and microbial-derived products, thereby inducing aberrant immune activation. The tight regulation of the Treg/Th17 balance by healthy host–gut interactions is key to prevent this aberrant immune activation [37,39]. 

Given the influence of the microbiome on the immune system, it is not surprising that gut dysbiosis leads to alterations of both local and systemic responses [36]. 

Through the translocation of proinflammatory molecules from the gut microbiota into the blood (metabolic endotoxemia), gut microbes contribute to the onset of low-grade inflammation and an altered fat metabolism [40,41]. 

Recent evidence also points towards the regulatory role of gut microbes with regard to fat mass development [42]. Obesity has shown to be associated with an over-representation of saccharolytic gut microbiota (obesity-associated dysbiosis) that facilitate augmented food digestion, leading to higher energy consumption and increased fat deposition, thus contributing to obesity development [36]. Moreover, when the microbiome of an obese individual is transplanted into mice, these mice show increased adiposity compared to mice that were transplanted with the microbiome of a lean individual [43].

Aging also affects the gut microbiome [44]. An increase in chronological age is associated with an increase in phylogenetic richness of the gut microbiota, while biological age results in overall decreased richness. In addition, it has been reported that a certain group of bacteria associated with frailty increases with biological age. As biological age increases, gut dysbiosis increases. These dysbiotic changes in the aging gut provoke proinflammatory innate immunity and other pathological conditions [45]. 

It is well established that infectious agents can cause cancer, such as *Helicobacter pylori* causing gastric cancer or an oncogenic virus such as human papilloma virus or hepatitis B virus. However, many other cancers are associated with gut dysbiosis. Colorectal cancer (CRC) is probably the most well-studied cancer type in this regard [36]. It has been shown that CRC patients have reduced levels of butyrate-producing bacteria in their gut microbiota and it is speculated that the reduction of colonic butyrate could be responsible for the impaired immune surveillance and potentiation of tumorigenesis [46,47]. Very recently, the urinary microbiome profile was found to differ between prostate cancer patients and patients with benign diseases, which could potentially indicate that urinary microbiome profile might be used as a biomarker of prostate cancer [48].

## 3. Impact of Host Factors on Cancer Immunotherapies

### 3.1. Age 

The assessment of the effects of aging on cancer immunotherapy effectiveness has been hampered by the fact that elderly individuals are under-represented in clinical trials. Moreover, since the selection criteria for clinical trials are very stringent, the participating patients do not represent the whole elderly population [49,50,51,52,53].

Because of the limited enrollment of older patients in clinical trials, subgroup analyses of single trials do not have enough statistical power to draw firm conclusions. Recently, three different meta-analyses were performed to investigate whether age differences play a role in the efficacy of ICB based on a large amount of clinical data [54,55,56]. One report focused only on programmed death-1 (PD-1) or PD-L1 inhibitors, a second report focused on cytotoxic T lymphocyte antigen-4 (CTLA-4) and PD-1 blocking agents, and the third report investigated CTLA-4, PD-1 or PD-L1 inhibitors. All three studies covered trials of various tumor types. An age cut-off of 65 years was used to define old versus young patients. The clinical outcome parameters were overall and progression-free survival. Two of these meta-analyses demonstrated no difference in the efficacy of ICB between young and older patients and one meta-analysis actually showed that older patients benefited more from ICB than younger patients [54,55,56]. A higher benefit from ICB in older patients might be related to the increased levels of memory T cells in older individuals, since Zuazo et al. recently described that patients with a high proportion of memory CD4 T cells before commencing treatment identify as potential responder patients to PD-1 blockade [57]. Additionally, a recent study by Kugel et al. found that the intratumoral CD8/Treg ratio of aged melanoma patients treated with anti-PD-1 significantly increased compared to younger patients, which might also explain the benefit of older patients [58]. Furthermore, since aging is associated with increased PD-1 expression in T cells, it might be intuitive that these patients would better respond to PD-1 blockade.

Concerning other immunotherapeutic modalities, data from clinical trials reporting on efficacy in elderly patients is limited. For cancer vaccines, Gravekamp and Jahangir reviewed the literature on cancer vaccination in mice, showing that cancer vaccination is less effective at old than at young age, but that additional stimulation of the innate immune system (NK cells, NKT cells, and MDSCs) improve cancer vaccine efficacy in old mice [59]. 

Thus, the effect of aging might be dependent on the type of immunotherapy used. Moreover, it is not clear whether the positive results of ICB in patients older than 65 years also holds true for patients >75 years. However, since these patients aged >75 years constitute more than 25% of newly diagnosed cases each year [5], it is warranted to include more of these patients in clinical trials. An important parameter to take into account is that the arbitrary age cut-off might not be sufficient to characterize older patients. Indeed, the older patients that participate in clinical trials most likely do not represent the whole elderly population, since they are extremely selected due to the strict inclusion criteria used in clinical trials, thereby excluding patients with low performance status and several comorbidities. Therefore, specific studies should be performed to assess immunotherapy effectiveness in older patients with cancer, using a physiological age that takes into account the geriatric assessment and biomarkers of aging and immunosenescence, in order to better understand which subset of this growing population of cancer patients will benefit from a certain immunotherapy. Concerning toxicity of immunotherapy, scarce data up to now have not revealed major differences in the adverse event (AE) profile between patients younger and older than 65 years. However, patients treated with anticoagulants are at increased risk for gastrointestinal hemorrhage. Thus, future studies should also focus on the tolerance and toxicity of immunotherapies in elderly patients, which might be important in individuals with several comorbidities.

### 3.2. Obesity

The effects of obesity on the efficacy of cancer immunotherapies have been somewhat conflicting and may indicate that outcomes may be dependent on the type of immunotherapy. A very recent paper on PD-1 blockade across multiple species and tumor models showed that, despite the heightened immune dysfunction and tumor progression in obese patients, paradoxically, obesity was associated with greater anti-tumor efficacy and survival. However, intuitively it makes sense that the PD-1 associated T cell dysfunction in obesity renders these tumors more sensitive to PD-1 blockade [60]. A retrospective study in patients with metastatic melanoma that were treated with immune checkpoint blockers (CTLA-4 or PD-1/PD-L1 targeting antibodies) showed that obesity was associated with improved progression-free and overall survival, an effect only observed in male patients [61]. 

In contrast, in patients with high-risk, non-muscle-invasive bladder cancer treated with bacillus Calmette–Guérin immunotherapy, overweightness and obesity were associated with an increased risk of disease progression [62]. In a diet-induced obesity (DIO) mouse model of renal cell carcinoma, treatment with adenovirus-encoded TNF-related apoptosis-inducing ligand TRAIL (AdTRAIL) plus CpG 1826 failed to decrease the tumor burden in contrast to the response observed in mice with normal weight [63]. Murphy et al. showed that treatment with anti-CTLA-4 or AdTRAIL+CpG prolonged survival in lean and ob/ob obese mice, but not in DIO mice and found that this was due to the elevated leptin serum levels in DIO mice as compared to normal weight and ob/ob mice. These data indicate that leptin neutralization could serve as a means of increasing immunotherapy effectiveness in obese cancer patients [64]. 

Even less data are available on the effect of obesity on immunotherapy toxicity. Mirsoian et al. noted the induction of a systemic cytokine storm resulting in lethality upon treatment of obese mice with anti-CD40 and IL-2, which was not seen in lean mice. They observed this adiposity-related toxicity in different models: aged ad-libitum-fed mice, ob/ob mice, and DIO mice. Blocking TNF during treatment prevented the toxic effects in obese mice [65]. To our knowledge, stratification of patients based on BMI has not been reported. However, it would be of interest to retrospectively stratify patients from clinical trials using different types of immunotherapies based on BMI to determine whether BMI is correlated to immunotherapy toxicity.

### 3.3. Microbiome

Two main observations have demonstrated the importance of the microbiota on the response to cancer immunotherapy in humans. First, antibiotics treatment was shown to compromise the efficacy of PD-1 blockade in mouse tumor models and cancer patients [66,67]. Second, fecal microbiota transplantation (FMT) from cancer patients who responded to ICB into germ-free or antibiotic-treated mice ameliorated the antitumor effects of ICB, whereas FMT from non-responding patients failed to do so [66,68]. For PD-1 blockade, specifically, it has been shown that responder patients have a higher diversity in their gut microbiota and certain bacterial strains are associated with response [37,69,70,71]. Both in mice and humans, the role of microbiota in the toxicity induced by ICB has also been shown, since certain bacterial strains have been shown to have a protective role [37,69,70]. Thus, there are clearly bacterial taxa that are associated with response and toxicity to ICB, but there does not seem to be great overlap among bacterial taxa associated with response, although some taxa that are implicated with either response or toxicity are related to each other. However, differences observed in separate studies may also be related to the techniques used, highlighting the importance of developing standardized techniques for microbiome analysis. These studies also did not take into account the influence of aging, obesity, and other lifestyle factors [39]. 

While previous studies focused on ICB, recent data also suggest a role for the intestinal microbiota in mediating the response to chimeric antigen receptor T cells (CAR-T cells) and propose that the baseline microbiota may correlate with efficacy and toxicity. Interestingly, the microbiota identified in this study differed from the bacterial species that have been found to promote anti-tumor immunity to ICB [72]. 

The gut microbiome might also be impacted in the immunotherapeutic effects of low-dose cyclophosphamide, since it was shown that cyclophosphamide induces an intestinal dysbiosis-mediated increase in gut leakiness, leading to translocation of Gram-positive bacteria to lymphoid tissue, and causing activation of interferon IFNγ^+^ Th17 cells [73]. Similarly, the immunotherapeutic efficacy of CpG-oligodeoxynucleotides therapy seems to be dependent on TNF production by microbiome-primed tumor-associated myeloid cells [74]. 

Currently, clinical trials are being performed in an attempt to modulate the microbiome. Probiotics are widely used to reduce the side effects of anti-cancer treatments, such as diarrhea or mucositis, for which species in the *Lactobacillus* and *Bifidobacteria* genera are most frequently used. One clinical trial (NCT03829111) is evaluating whether the *Clostridium butyricum* CBM 588 probiotic strain, in combination with nivolumab and ipilimumab, is able to influence the gut microbiome and clinical efficacy in metastatic renal cell carcinoma. Another trial (NCT03891979) is evaluating whether the combination of pembrolizumab with antibiotics (ciprofloxacin and metronidazole) during the preoperative period leads to changes in immune activation in pancreatic tumor tissue. In the MIMIc study (NCT03772899), the investigators will combine FMT with pembrolizumab or nivolumab in advanced melanoma using fecal material from a healthy donor. In addition to assessing the safety of the combination, the investigator will also study the effect of FMT on the immune system and microbial ecosystem of the gut. As an alternative to FMT, microbial ecosystem therapeutics (METs) were developed as a new treatment approach, which consists of a defined mixture of pure live cultures of intestinal bacteria isolated from a stool sample of a healthy donor. The NCT03686202 study was designed to assess the safety, tolerability, and engraftment of MET-4 strains when given in combination with ICB. Finally, an interesting approach is investigated in NCT03817125, wherein melanoma patients will undergo an antibiotic or antibiotic placebo treatment lead-in to prime the gut microbiome for engraftment of the oral microbiome study intervention. The oral microbiome therapy (SER-401) consists of a mixture of live bacteria, similar to the one seen in patients who responded to cancer immunotherapy. The SER-401 therapy will be given together with nivolumab and patients will be assessed for safety, changes in the microbiome, changes in the percentage of tumoral CD8 T cells, and antitumor activity.

Thus, overall, most of the evidence for the implications of the microbiome in cancer immunotherapy effectiveness is available for ICB, with limited evidence for other types of immunotherapy. Clearly, the available data show that the microbiome is implicated in response and toxicity to cancer immunotherapy, but further research is needed to clearly define microbiome signatures predictive of efficacy and/or toxicity. 

## 4. Implications for Future Research

**Figure 2:**
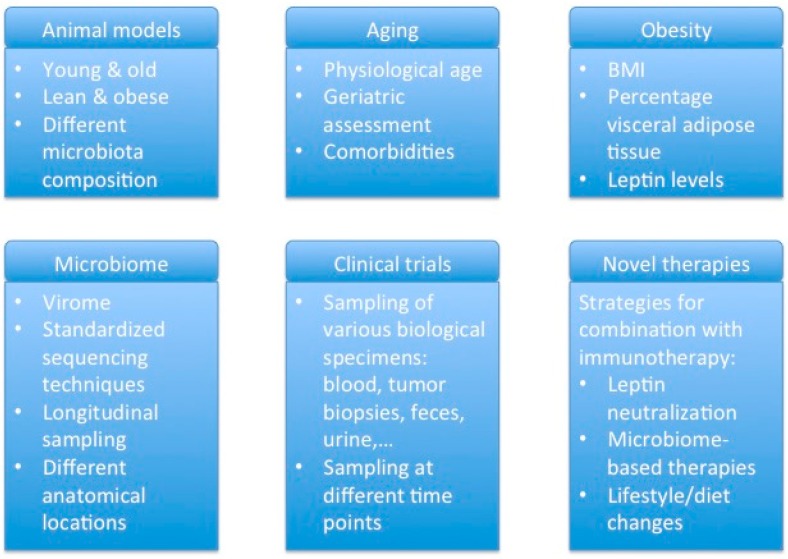
Suggestions for future research/clinical studies that aim to evaluate the effects of aging, obesity or the microbiota.

Investigation of the influence of aging, obesity, and the microbiome on the outcome of cancer immunotherapy has been hampered by the lack of reliable animal models that recapitulate the complexity of humans. Indeed, most research has been performed in young, lean, and inbred mice. Since these variables have an impact on immunity, this may account in part for the discrepancies observed between immunotherapy effectiveness and toxicity between mouse models and cancer patients. This is exemplified by the fact that mice from different providers are colonized by distinct microbiota and show different responses to ICB [75]. Therefore, in order to be able to better extrapolate mouse data to humans, cancer immunotherapies should be systematically tested in different mouse models, young and old, lean and obese, and with a different microbiota composition (Figure 2) [76,77].

The available clinical data clearly indicate the potential of cancer immunotherapies in elderly and obese individuals. Given the impact of the microbiome on efficacy and toxicity and since both aging and obesity considerably influence the composition of the microbiota, it is of the utmost importance to incorporate these factors in the design of clinical trials. To assess the cancer immunotherapy response in elderly individuals, we recommend the set-up of specific trials enrolling patients from different age categories. Herein, it is important to not only focus on chronological age, but rather on physiological age that takes into account the geriatric assessment and comorbidities, since these factors might have an impact on toxicity. For studies on obesity, not only the BMI should be taken into account, but also the percentage of visceral adipose tissue and leptin levels in blood should be assessed. Clinical trials should be accompanied by sampling of blood, biopsies, feces, and urine at different time points if possible, to be able to identify biomarker signatures associated with response or toxicity (Figure 2). 

These recent findings suggest a strong influence of the microbiome on cancer immunotherapy. However, our understanding of the (gut) microbiome is still very preliminary. For example, most studies until now focused on the bacterial component of the microbiome. However, it is likely that another important component of the microbiome, namely the virome, can also exert effects. Therefore, it is urgently needed to broaden microbiome studies to get a complete and clear understanding of the interaction between our microbiome and cancer immunotherapy. This implies the need for better virome sequencing techniques, standardization of microbiome sequencing techniques across studies, and the execution of large-scale longitudinal clinical trials (Figure 2). These studies will allow the identification of microbiome signatures associated with response or toxicity to a certain immunotherapy and contribute to better patient selection.

Finally, an improved understanding of the changes in the immune system and the microbiome with aging and obesity will also have implications for the development of therapeutic strategies to enhance response or reduce toxicity to a certain immunotherapy in a defined patient population. For instance, leptin neutralization may improve cancer vaccination in obese individuals. Likewise, the knowledge about microbiome changes induced by aging or obesity might pave the way towards therapeutic strategies to restore the altered microbiota towards a favorable composition for cancer immunotherapy. Microbiome-based therapies, such as fecal microbiota transplantation, probiotics, and prebiotics, as well as lifestyle/diet changes are emerging as adjuncts to cancer immunotherapy treatment (Figure 2).

## 5. Conclusions

Cancer immunotherapy has now become one of the pillars of cancer treatment. However, it is clear that not all patients respond, and that sometimes, severe toxicities can occur. Furthermore, different types of immunotherapy have a distinct mode of action. In order to be able to select the best type of immunotherapy for the right patient, we should take into account the natural variability among diverse patient populations. It is clear that host factors such as aging, obesity, and microbiota composition strongly affect immunity. Therefore, future studies should focus on unraveling the implication of these host variables on the outcome of distinct immunotherapies. In this way, we can develop biomarker signatures per type of immunotherapy to select for patients with the highest probability of response and the lowest risk of toxicity. Furthermore, these insights will also shed light on how manipulation of these host variables (e.g., through diet/lifestyle changes, probiotics) can be used as an adjunct to immunotherapy to restore alterations. 

On the one hand, these studies will enable better patient stratification, and on the other hand, we predict that these studies will allow cancer immunotherapy treatment of patients that would otherwise be excluded from this treatment option (e.g., aged or obese individuals with several comorbidities).
